# Identification of immune infiltration and cuproptosis-related molecular clusters in tuberculosis

**DOI:** 10.3389/fimmu.2023.1205741

**Published:** 2023-07-11

**Authors:** Sijun Li, Qian Long, Lanwei Nong, Yanqing Zheng, Xiayan Meng, Qingdong Zhu

**Affiliations:** ^1^ Infectious Disease Laboratory, The Fourth People’s Hospital of Nanning, Nanning, China; ^2^ Department of Clinical Laboratory, The Fourth People’s Hospital of Nanning, Nanning, China; ^3^ Department of Tuberculosis, The Fourth People’s Hospital of Nanning, Nanning, China

**Keywords:** tuberculosis, cuproptosis, molecular clusters, immune infiltration, machine learning model

## Abstract

**Background:**

Tuberculosis (TB) is an infectious disease caused by Mycobacterium tuberculosis (Mtb) infection. Cuproptosis is a novel cell death mechanism correlated with various diseases. This study sought to elucidate the role of cuproptosis-related genes (CRGs) in TB.

**Methods:**

Based on the GSE83456 dataset, we analyzed the expression profiles of CRGs and immune cell infiltration in TB. Based on CRGs, the molecular clusters and related immune cell infiltration were explored using 92 TB samples. The Weighted Gene Co-expression Network Analysis (WGCNA) algorithm was utilized to identify the co-expression modules and cluster-specific differentially expressed genes. Subsequently, the optimal machine learning model was determined by comparing the performance of the random forest (RF), support vector machine (SVM), generalized linear model (GLM), and eXtreme Gradient Boosting (XGB). The predictive performance of the machine learning model was assessed by generating calibration curves and decision curve analysis and validated in an external dataset.

**Results:**

11 CRGs were identified as differentially expressed cuproptosis genes. Significant differences in immune cells were observed in TB patients. Two cuproptosis-related molecular clusters expressed genes were identified. Distinct clusters were identified based on the differential expression of CRGs and immune cells. Besides, significant differences in biological functions and pathway activities were observed between the two clusters. A nomogram was generated to facilitate clinical implementation. Next, calibration curves were generated, and decision curve analysis was conducted to validate the accuracy of our model in predicting TB subtypes. XGB machine learning model yielded the best performance in distinguishing TB patients with different clusters. The top five genes from the XGB model were selected as predictor genes. The XGB model exhibited satisfactory performance during validation in an external dataset. Further analysis revealed that these five model-related genes were significantly associated with latent and active TB.

**Conclusion:**

Our study provided hitherto undocumented evidence of the relationship between cuproptosis and TB and established an optimal machine learning model to evaluate the TB subtypes and latent and active TB patients.

## Introduction

Tuberculosis (TB) is an infectious disease caused by Mycobacterium tuberculosis (Mtb) infection (1). A clinical diagnosis of TB is usually established based on the following criteria (2–5): 1) a positive tuberculin skin test (TST). 2) abnormal chest radiographs: a single lesion with enlargement of the draining lymph nodes in lung, a single lesion with unremarkable lymph nodes and multiple secondary tubercles in lung and miliary lesions throughout the lung,etc. 3) clinical evidence of current disease: chronic productive cough, hemoptysis, low-grade fever, night sweats, loss of appetite, malaise, fatigue and weight loss,etc. 4) A positive culture of Mtb (the most important technique to diagnose TB). Currently, acid-fast bacilli (AFB) testing and Mtb culture remain the mainstay for detecting mycobacterium TB. AFB takes less time but being less accurate (6, 7). Current evidence suggests that the culture of pathogenic microorganisms is the gold standard for identifying TB with specificity rates greater than 99% (5). However, false-positive cultures for Mtb are not rare due to contamination of clinical devices, clerical errors, and laboratory cross-contamination (8). There is a possibility of missed diagnosis and misdiagnosis of TB. Hence, identification of TB is a huge challenge.

The number of people with undiagnosed and untreated TB has grown, resulting first in an increased number of TB deaths and more community transmission of infection and then, with some lag-time, increased numbers of people (9). In 2021, there were an estimated 1.6 million deaths (9). Patients infected with Mtb may experience physical stress. Alexandria Jones-Patten found that depressive symptoms were reported in 26.1% TB patients and anxiety symptoms were reported in 47.2% TB patients (10). The Mtb infection leads to different clinical characteristics of symptoms, which affects the quality of life of patients and brings a heavy burden to families and society (4, 11). About half of TB patients and their households face catastrophic total costs due to TB disease (9). Therefore, it is of great clinical significance to accurately identify the biomarker of TB at the molecular level and establish multivariate prediction models.

A deeper understanding of the underlying mechanism of TB is warranted to identify the biomarker associated with TB. Copper, a critical element for life, is a catalytic or structural cofactor essential to various biological processes, including mitochondrial respiration, antioxidant defense, and biosynthesis (12). However, dysregulation of copper homeostasis has been proven to be associated with diseases (13). Previous research suggested that copper is involved in the pathogenesis of TB. Alex G. Dalecki et al. suggested that the copper ions could kill Mtb (14). G. Mohan found that the serum copper levels of TB patients were decreased after antitubercular treatment (15). Gnogbo Alexis Bahi et al. demonstrated that the copper was closely associated with multidrug-resistant TB (16). Copper appears to play an important role in TB, but the underlying mechanism remains unclear. Cuproptosis was proposed as a novel cell death mechanism (17). Excess copper accumulation triggers the destruction of iron-sulfur cofactors and stimulates destructive reactive oxygen species produced by copper-driven iron-death reactions, ultimately causing cell death (18). Furthermore, copper binds to the acylated component of the tricarboxylic acid (TCA)cycle, causing acylated protein aggregation and depletion of iron-sulfur cluster proteins, leading to cell death (17). Therefore, it is highly conceivable that cuproptosis is closely related to the development of TB. Further illustrating the molecular characteristics of cuproptosis-related genes (CRGs) may explain the heterogeneity of TB and provide a new perspective for the clinical diagnosis and treatment of TB.

In this study, the differential expression analysis of CRGs and immune signatures was conducted between normal and TB individuals. A predictive model was developed to identify patients with distinct molecular clusters by comparing different machine-learning algorithms. The correlation between model-related genes with latent TB and active TB was investigated in an external TB cohort. Finally, our study may provide novel insights into the prediction of TB and the differentiation between latent and active TB.

## Materials and methods

### Experimental design

**Figure 1:**
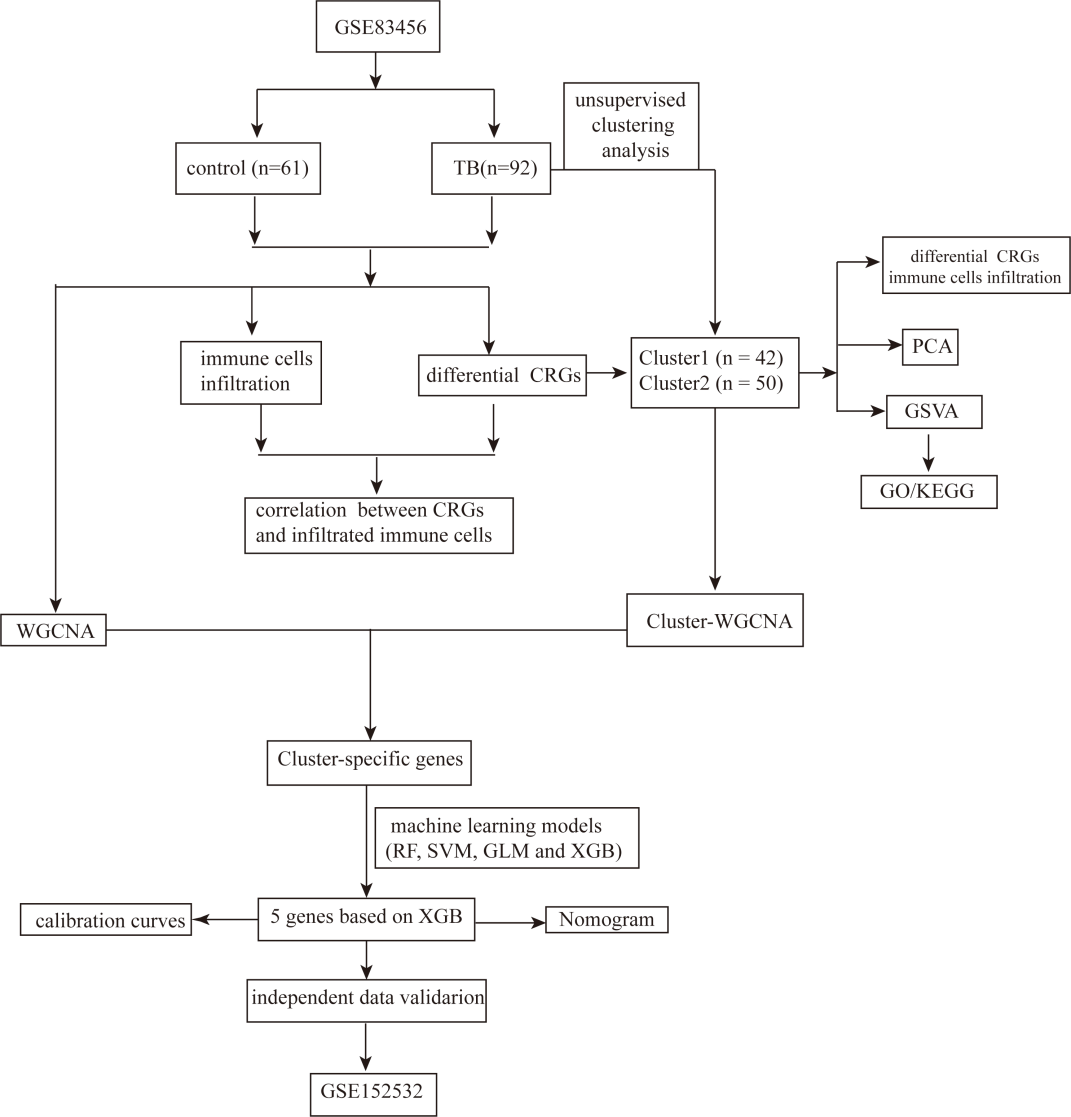
Schematic representation of the experimental design used to investigate the role of CRGs in TB.

The experimental design is illustrated in **Figure 1**.

### Data acquisition and preprocessing

Two microarray datasets (GSE83456 and GSE152532) were obtained from the Gene Expression Omnibus (GEO) database (www.ncbi.nlm.nih.gov/geo). Perl programming language was employed to preprocess the two microarray datasets. Microarray datasets were merged with probe platform.The GSE83456 dataset (GPL10058 platform, PMID: 27706152), including 61 blood samples from healthy people (control group) and 92 blood samples from TB patients (TB group), was selected for preliminary analysis. Diagnosis of TB was based on the following: positive mycobacterial culture result from the site of disease; or caseating granuloma on biopsy and/or clinical/radiological features consistent with TB and a good response to therapy (19). The GSE152532 dataset (GPL10058 platform, PMID: 34555657), including 11 blood samples from healthy people and 136 blood samples from TB patients, was selected for validation. The blood samples from TB patients in GSE152532 dataset contained 111 latent TB samples and 25 active TB samples. Latent TB was confirmed in participants with a positive interferon gamma release assay(IGRA) and the absence of clinical and radiographic signs of ATB or any other significant co-morbidity. Active TB was defined as either a suspected (based on clinical suspicion and radiological and/or histological evidence) or microbiologically confirmed (by Mtb culture) new diagnosis of pulmonary or extrapulmonary TB disease, in the absence of any other significant co-morbidity (20).The cuproptosis-related genes in the Molecular Signature Database (MsigDB) v7.0 database (http://www.gsea-msigdb.org/gsea/msigdb/) were combined with gene sets relevant to cuproptosis from a prior study (21). R programming language (version 4.1.3) was employed to conduct this study.

### Identification of differentially expressed CRGs in TB

The R package “limma” was utilized to identify differentially expressed CRGs (threshold P<0.05) from the GSE83456 dataset. Subsequently, the R packages “ggpubr” and “pheatmap” were utilized to generate box plots and heat maps, respectively. The differential CRGs were correlated by the R package “corrplot “ to explore correlations between genes.

### Assessment of immune cell infiltration

The common method for studying cell heterogeneity, such as flow cytometry, rely on a limited repertoire of phenotypic markers. However, tissue disaggregation before flow cytometry can lead to lost or damaged cells, altering results (22). Aaron M Newman et al. presented cell-type identification by estimating relative subsets of RNA transcripts (CIBERSORT), a computational approach that accurately resolves relative fractions of diverse cell subsets in GEPs from complex tissues (23). CIBERSORT (https://CIBERSORT.stanford.edu//) is an analytical tool from the Alizadeh Lab and Newman Lab to impute gene expression profiles and provide an estimation of the abundances of member cell types in a mixed cell population, using gene expression data. Based on the gene expression data of the GSE83456 dataset, the CIBERSORT algorithm and Leukocyte signature matrix (LM22) were used to estimate the relative abundance of 22 immune cells in each sample. LM22 is a gene matrix that contains 547 white blood cells characteristic genes to differentiate 22 types of immune cells, including myeloid subgroup, Natural killer (NK) cells, naive and memory B-cells, and seven types of T-cell (24). Monte Carlo sampling was used to obtain the inverse folded product p-value of each sample. Only the samples with p-value < 0.05 were considered accurate immune cell components. The sum of the 22 immune cells in each sample was 1.

### Correlation analysis between CRGs and infiltrated immune cells

The correlation coefficient between CRGs and the characteristics of relevant immune cells was analyzed to further demonstrate the correlation between CRGs expression and the relative percentage of immune cells. Spearman correlation analysis was conducted, and a p-value less than 0.05 indicated a significant correlation. Finally, the results were visualized using the R package “corrplot”.

### Clustering of TB patients

Based on the expression profile of CRGs, the R package “ConsensusClusterPlus” was utilized to apply the unsupervised clustering analysis. The 92 TB samples were classified into clusters using the k-means algorithm with 1,000 iterations. k values are defined from 1 to 9 to generate different subtypes. The optimal number of clusters was selected according to the cluster consensus score. The principal component analysis (PCA) was used to visualize the distribution of the clusters.

### Gene set variation analysis analysis

GSVA contributes to the current need of Gene set enrichment methods for RNA-seq data (25). GSVA is an open source software package for R which forms part of the Bioconductor project and can be downloaded at http://www.bioconductor.org. The R package “GSVA” was used for GSVA enrichment analysis to elucidate the differences of enriched gene sets among different CRGs clusters. The gene matrix transposed (gmt) file,including “ c2.cp.kegg.v2022.1.Hs.symbols.gmt” and “ c5.all.v2022.1.Hs.symbols.gmt”, were downloaded from the MSigDB website database for further GSVA analysis. The R package “limma” was utilized to identify differentially expressed pathways and biological functions by comparing GSVA scores between different CRGs clusters. A p-value < 0.05 was statistically significant.

### Weighted gene co-expression network analysis

Weighted correlation network analysis (WGCNA) can be used for finding clusters (modules) of highly correlated genes, for summarizing such clusters using the module eigengene or an intramodular hub gene, for relating modules to one another and to external sample traits (using eigengene network methodology), and for calculating module membership measures (26).The R packages “WGCNA” was utilized to identify the co-expression modules. The top 25% genes with the highest variance were used for WGCNA analysis to guarantee the accuracy of quality results. The optimal soft power was selected to construct the weighted adjacency matrix, which was further transformed into a topological overlap matrix (TOM). When the minimum module size was set to 100, the TOM dissimilarity measure (1-TOM) based on the hierarchical clustering tree algorithm was used to obtain the module. Each module was assigned a random color. Modular characteristic genes represent the overall gene expression profile of each module. Gene significance (GS) represented the correlation between genes and clinical phenotypes. The relationship between modules and disease status was reflected by module significance (MS). MS, defined as the mean of the gene significance values of all genes within a module, represents the correlation between module genes and traits

### Construction of predictive model based on multiple machine learning algorithms

Based on two different CRGs clusters, the R package “caret” was applied for establishing machine learning models, including random forest (RF), support vector machine (SVM), generalized linear model (GLM), and eXtreme Gradient Boosting (XGB). The R package “pROC” was utilized to visualize the area below the ROC curve. The five genes with the lowest dropout-loss values were considered as top five variables. Therefore, the optimal machine learning model was determined, and the top five variables were identified as the key predictive genes associated with TB. ROC curve analysis was utilized to verify the diagnostic value of the diagnostic model in the GSE152532 dataset. Finally, based on the key predictive genes of TB, Spearman correlation analysis was performed in the GSE152532 dataset to explore the associations between prediction model-related genes with latent TB and active TB. A p-value < 0.05 was statistically significant.

### Construction and validation of a nomogram

The the top five variables were considered as the predictors. Based on the predictors, a nomogram was constructed to identify TB clusters by the R package “rms”. Each predictor was attributed a score, and the “total score” was obtained by summing the scores of all predictors. Calibration curve analysis and decision curve analysis (DCA) were applied to evaluate the predictive performance of the nomogram.

### Statistical analysis

R Programming Language (version 4.1.3) was used for data analysis and statistical analyses. Bilateral wilcoxon tests were utilized to evaluate statistical differences between the two groups. Spearman correlation, calculated by “cor.test” function,was applied to analyze the relationship between the expression level of cuproptosis-related genes and immune cells. A p-value < 0.05 was statistically significant.

## Result

### Identification of CRGs clusters in TB

**Figure 2:**
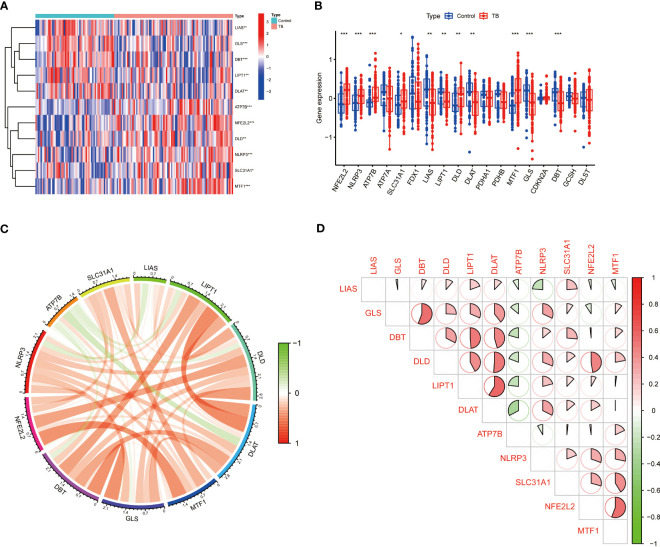
Identification of differentially expressed CRGs in patients with TB. **(A)** The expression levels of CRGs were presented in the heatmap. A total of 11 CRGs were identified as differentially expressed cuproptosis genes. **(B)** The expression levels of 11 CRGs were exhibited between control and TB groups in boxplots. The expressions of NFE2L2, NLRP3, ATP7B, SLC31A1, MTF1, and DLD were upregulated; LIAS, LIPT1, DLAT, GLS, and DBT were downregulated in TB patients. **(C, D)** Correlation analysis of 11 differentially expressed CRGs. Red and green colors respectively represent positive and negative correlations. Significant positive correlations were found between NFE2L2 and MTF1, LIPT1 and DLAT, DLD and DLAT, as well as GLS and DBT (*p<0.05, **p<0.01, ***p<0.001).

To clarify the biological function of CRGs in the occurrence and development of TB, the GSE83456 dataset was utilized to assess the differential expression of CRGs between TB and healthy people. A total of 11 CRGs were identified as differentially expressed cuproptosis genes (**Figure 2A**). The expressions of NFE2L2, NLRP3, ATP7B, SLC31A1, MTF1, and DLD were upregulated, while LIAS, LIPT1, DLAT, GLS, and DBT were downregulated in TB patients (**Figure 2B**). Subsequently, correlation analysis between these differentially expressed CRGs was performed to explore whether cuproptosis regulators participated in the progression of TB (**Figure 2C**). Among these genes, significant positive correlations were found between NFE2L2 and MTF1 (correlation coefficient, r= 0.56), LIPT1 and DLAT (correlation coefficient,r= 0.59), DLD and DLAT (correlation coefficient,r = 0.51), as well as GLS and DBT (correlation coefficient,r = 0.55) (**Figure 2D**).

### Immune infiltration in TB

**Figure 3:**
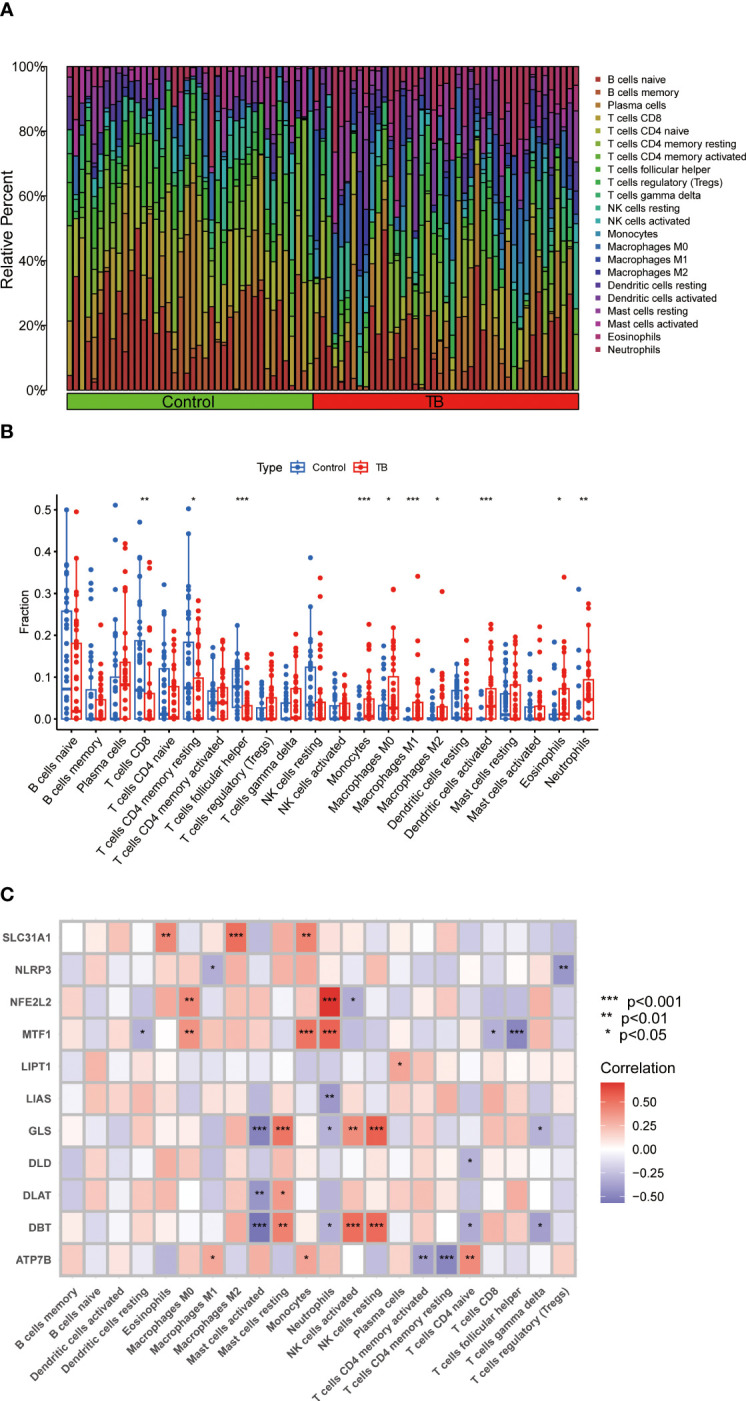
Analysis of immune cell infiltration in patients with TB. **(A)** CIBERSORT analysis revealed differences in the abundance of 22 infiltrating immune cell types between the TB and control groups. **(B)** The differences in immune infiltration between control and TB groups are shown in the boxplot. CD8+ T cells, resting memory CD4+ T cells, and follicular helper T cells were significantly decreased, while monocytes, M0, M1, and M2 macrophages, activated dendritic cells, eosinophils, and neutrophils were increased in the TB group **(C)** Correlation analysis between 11 differential CRGs and infiltrated immune cells. Resting dendritic cells, eosinophils, M0, M1, and M2 macrophages, activated mast cells, resting mast cells, monocytes, neutrophils, activated NK cells, resting NK cells, plasma cells, activated memory CD4+ T cells, resting memory CD4+ T cells, naïve CD4+ T cells, CD8+ T cells, follicular helper T cells, gamma delta T cells, and T cells regulatory (Tregs) were associated with CRGs. *p<0.05, **p<0.01, ***p<0.001.

An immune infiltration analysis was conducted to clarify immune differences between the TB and control groups. CIBERSORT analysis revealed differences in the abundance of 22 infiltrating immune cell types between the TB and control groups (**Figure 3A**). CD8+ T cells, resting memory CD4+ T cells, and follicular helper T cells were significantly decreased, while monocytes, M0, M1, and M2 macrophages, activated dendritic cells, eosinophils, and neutrophils were increased in the TB group (**Figure 3B**). Meanwhile, resting dendritic cells, eosinophils, M0, M1, and M2 macrophages, activated mast cells, resting mast cells, monocytes, neutrophils, activated NK cells, resting NK cells, plasma cells, activated memory CD4+ T cells, resting memory CD4+ T cells, naïve CD4+ T cells, CD8+ T cells, follicular helper T cells, gamma delta T cells, and T cells regulatory (Tregs) were associated with CRGs (**Figure 3C**). These results suggest that CRGs are key factors in regulating molecular and immune-invasive states in TB patients.

### CRGs-clusters in TB

**Figure 4:**
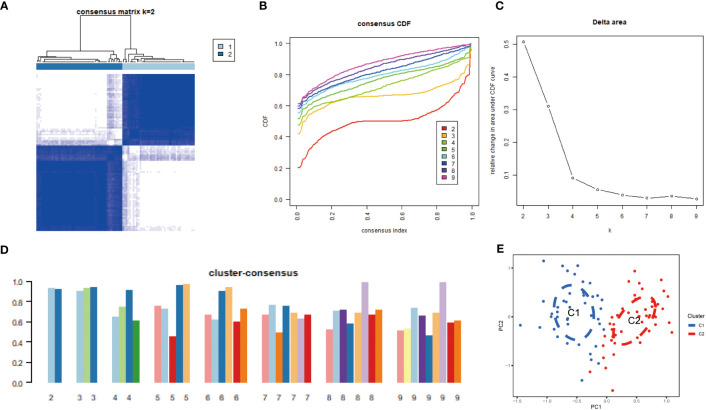
Identification of molecular subtypes associated with cuproptosis in TB. **(A)** The cluster number is most stable when the k value is set to 2. **(B)** the CDF curve fluctuates within the minimum range of the consensus index of 0.2 to 0.6. **(C)** the area under the CDF curve shows the difference between the two CDF curves. **(D)** when k = 2, the concordance score of each subtype was the highest(k=2). **(E)** PCA showed significant differences between the two clusters. The 92 TB patients could be divided into Cluster 1 (n = 42) and Cluster 2 (n = 50), which were significantly different.

Based on the expression of 11 CRGs, 92 TB samples were grouped using a consensus clustering algorithm. The optimal number of clusters was observed when the k value was set to 2 (k = 2), and the CDF curve fluctuated within the minimum range of the consensus index of 0.2 to 0.6 (**Figures 4A, B**). At k = 2 ~ 9, the area under the CDF curve exhibited differences between two CDF curves (k and k-1) (**Figure 4C**). At k = 2, the concordance score of each subtype was the highest (**Figure 4D**). PCA analysis revealed that the 92 TB patients could be divided into Cluster 1 (n = 42) and Cluster 2 (n = 50), which were significantly different (**Figure 4E**).

### Differentiation of CRGs and immune infiltration between the CRGs-clusters

**Figure 5:**
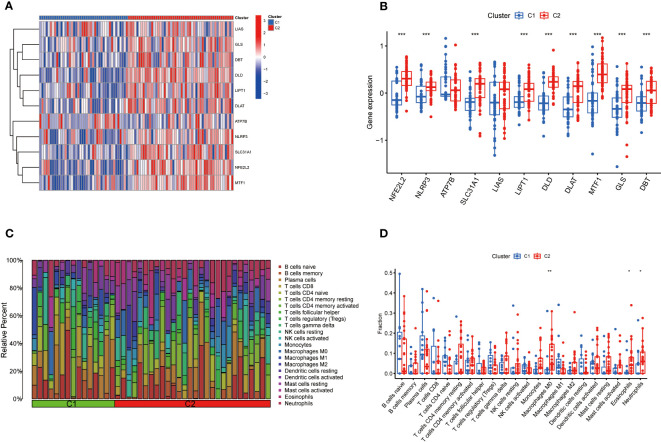
Comparison of CRGs expression and immune cell infiltration between molecular subtypes of TB. **(A)** Distinct CRGs expression profiles were observed between Cluster 1 and Cluster 2. **(B)**The expression of 13 CRGs between two clusters was presented in the boxplot. The expression of NFE2L2, NLRP3, SLC31A1, LIPT1, DLD, DLAT, MTF1, GLS, and DBT was significantly upregulated in Cluster 2 **(C)** The difference in the abundance of 22 infiltrating immune cell types between the two clusters. **(D)** The differences in immune infiltration between control and TB groups are shown in a boxplot. The abundance of M0 macrophages, eosinophils, and neutrophils was significantly increased. *p<0.05, **p<0.01, ***p<0.001.

The expression differences of 11 CRGs between Cluster 1 and Cluster 2 were comprehensively evaluated to explore the molecular characteristics between clusters. Distinct CRGs expression profiles were observed between Cluster 1 and Cluster 2 (**Figure 5A**). The expression of NFE2L2, NLRP3, SLC31A1, LIPT1, DLD, DLAT, MTF1, GLS, and DBT was significantly upregulated in Cluster 2(**Figure 5B**). In addition, the immune cell infiltration analysis showed significant differences in the immune microenvironment between Cluster 1 and Cluster 2 (**Figure 5C**). The abundance of M0 macrophages, eosinophils, and neutrophils was significantly increased (**Figure 5D**).

### Biological functions and pathway activities

**Figure 6:**
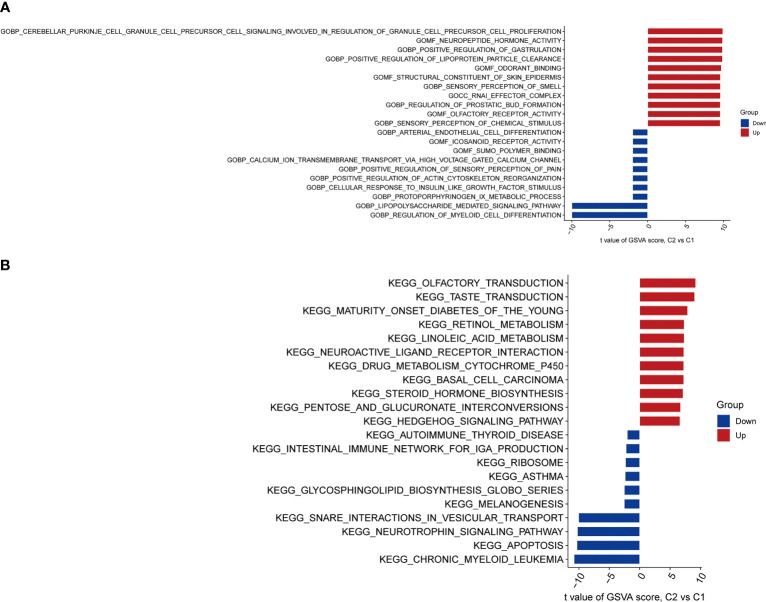
Biological functions and pathway activities between two CRG clusters. **(A)** Differences in biological functions between Cluster 1 and Cluster 2 samples ranked by t-value of GSVA method. Lipopolysaccharide-mediated signaling, regulation of myeloid cell differentiation, and protoporphyrinogen IX metabolic process were significantly enriched in Cluster 1. Cerebellar Purkinje cell granule cell precursor cell signaling involved in the regulation of granule cell precursor cell proliferation, neuropeptide hormone activity, and positive regulation of gastrulation were significantly enriched in Cluster 2.**(B)** Differences in hallmark pathway activities between Cluster 1 and Cluster 2 samples ranked by the t-value of the GSVA method. Chronic myeloid leukemia, apoptosis, and neurotrophin signaling pathways were significantly enriched in Cluster 1. Olfactory transduction, taste transduction, maturity-onset diabetes of the young, retinol metabolism, and neuroactive ligand-receptor interaction were significantly enriched in Cluster 2.

The pathway activities and biological functions associated with each group were identified by GSVA. The functional enrichment results showed that lipopolysaccharide-mediated signaling, regulation of myeloid cell differentiation, and protoporphyrinogen IX metabolic process were significantly enriched in Cluster 1 (**Figure 6A**). In contrast, cerebellar Purkinje cell granule cell precursor cell signaling involved in the regulation of granule cell precursor cell proliferation, neuropeptide hormone activity, and positive regulation of gastrulation were significantly enriched in Cluster 2 (**Figure 6A**). KEGG pathway analysis suggested that Chronic myeloid leukemia, apoptosis, and neurotrophin signaling pathways were significantly enriched in Cluster 1 (**Figure 6B**), while olfactory transduction, taste transduction, maturity-onset diabetes of the young, retinol metabolism, and neuroactive ligand-receptor interaction were significantly enriched in Cluster 2 (**Figure 6B**).

### Gene modules screening and co-expression network construction

**Figure 7:**
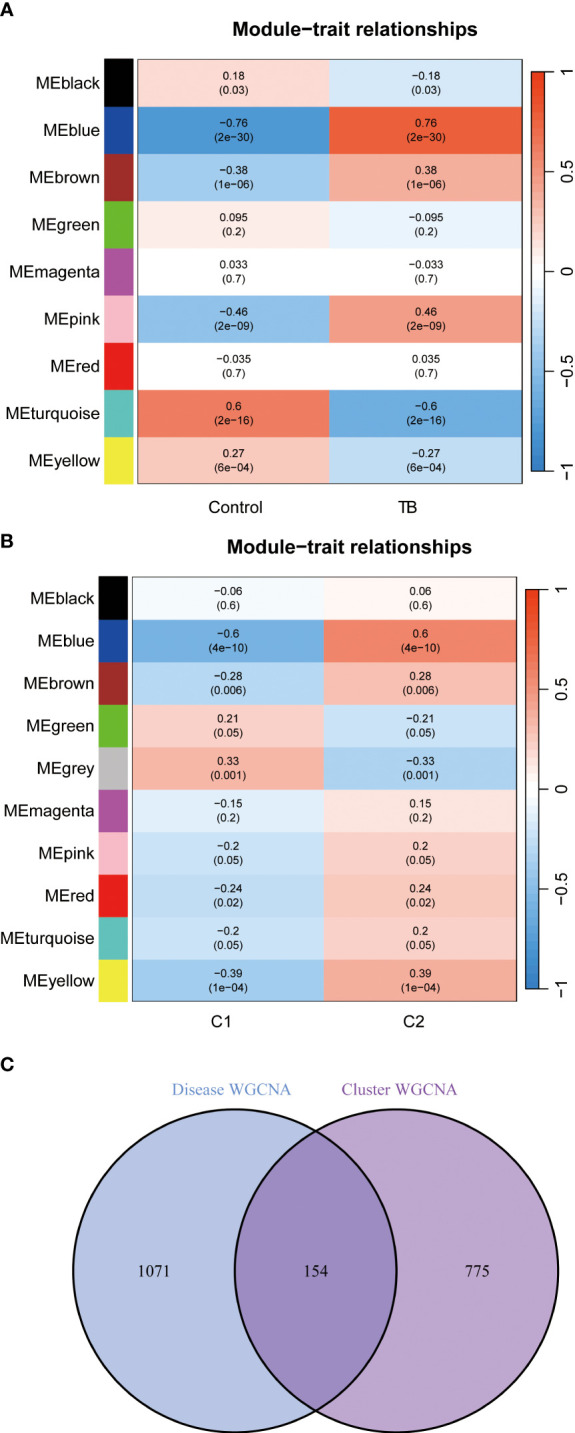
Identification of gene modules and co-expression networks associated with TB. **(A)** Correlation analysis between module eigengenes and clinical status in control and TB groups. The genes in the blue module exhibited the most significant relationship with TB. **(B)** Correlation analysis between module eigengenes and clinical status in the two clusters. Each row represents a module; each column represents a clinical status. Module-clinical features relationship analysis demonstrated a high correlation between the blue module and TB clusters. **(C)** Identification of the intersected genes of disease WGCNA and cluster-WGCNA. The intersection of genes in the two modules yielded 154 genes.

The WGCNA algorithm was applied to establish co-expression networks and modules in the normal population and TB patients to identify key gene modules associated with TB. The variance of each gene expression in the GSE83456 dataset was calculated to select the top 25% genes with the highest variance for further analysis. Ten co-expression modules with different colors were obtained by dynamic cutting algorithm, and a heat map of the topological overlap matrix (TOM) was generated. Among them, 1,225 genes in the blue module exhibited the most significant relationship with TB (**Figure 7A**). In addition, the WGCNA algorithm was conducted to analyze the key gene modules closely related to CRGs clustering. Module-clinical features (Cluster 1 and Cluster 2) relationship analysis demonstrated a high correlation between the blue module (929 genes) and TB clusters (**Figure 7B**). Finally, the intersection of genes in the two modules yielded 154 genes by the R package “Venn” (**Figure 7C**).

### Construction of machine learning models

**Figure 8:**
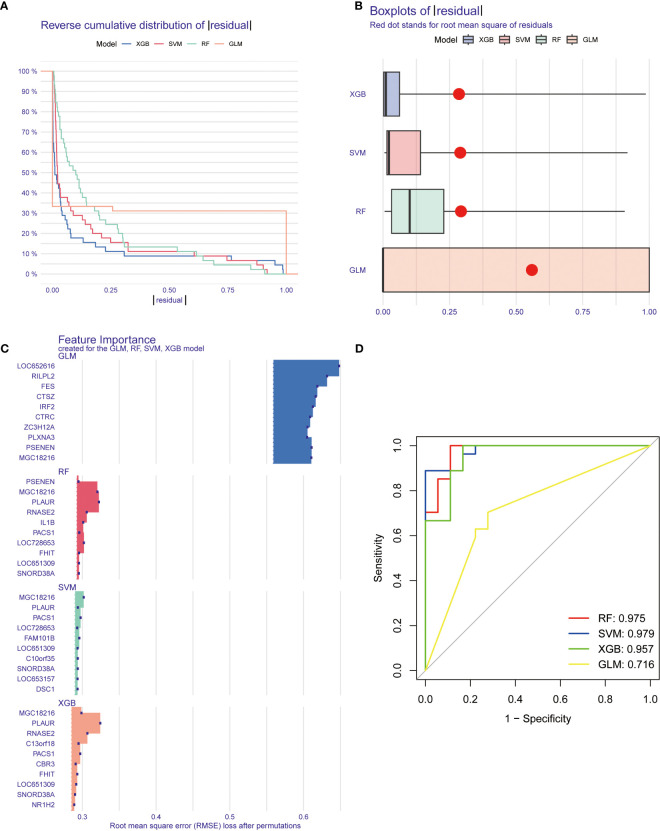
Construction and evaluation of machine learning models for predicting TB. **(A, B)** Residual distribution of each machine learning model. The residuals of XGB and SVM machine learning models were lower. **(C)** The important features in machine learning models. the genes of the top 15 features of each model were sequenced according to root mean square error. **(D)** ROC analysis of four machine learning models based on 5-fold cross-validation in the testing cohort. The areas under the AUC were obtained for the four models (RF, AUC=0.975; SVM, AUC=0.979; XGB, AUC=0.957; GLM, AUC=0.716).

Four machine learning models, including RF, SVM, GLM, and XGB, were constructed based on cluster-specific DEGs in the TB training cohort. The R package “DALEX” was applied to interpret the four models. The residual distribution of each model was plotted in the validation set. The residuals of XGB and SVM machine learning models were lower (**Figures 8A, B**). Subsequently, the genes of the top 15 features of each model were sequenced according to root mean square error (RMSE) (**Figure 8C**). Moreover, the discriminative performance of the four machine learning algorithms was evaluated by calculating receiver operating characteristic (ROC) curves based on 5-fold cross-validation in the training set (GSE83456 dataset) (**Figure 8D**). The areas under the ROC curve (AUC) were obtained for the four models (RF, AUC=0.975; SVM, AUC=0.979; XGB, AUC=0.957; GLM, AUC=0.716). Based on the residual and AUC, the XGB machine learning model demonstrated the best performance in distinguishing TB patients with different clusters. Finally, the top five genes (C13orf18, PACS1, MGC18216, RNASE2, and PLAUR) from the XGB model were selected as predictor genes for further analysis.

### Construction of nomogram model

**Figure 9:**
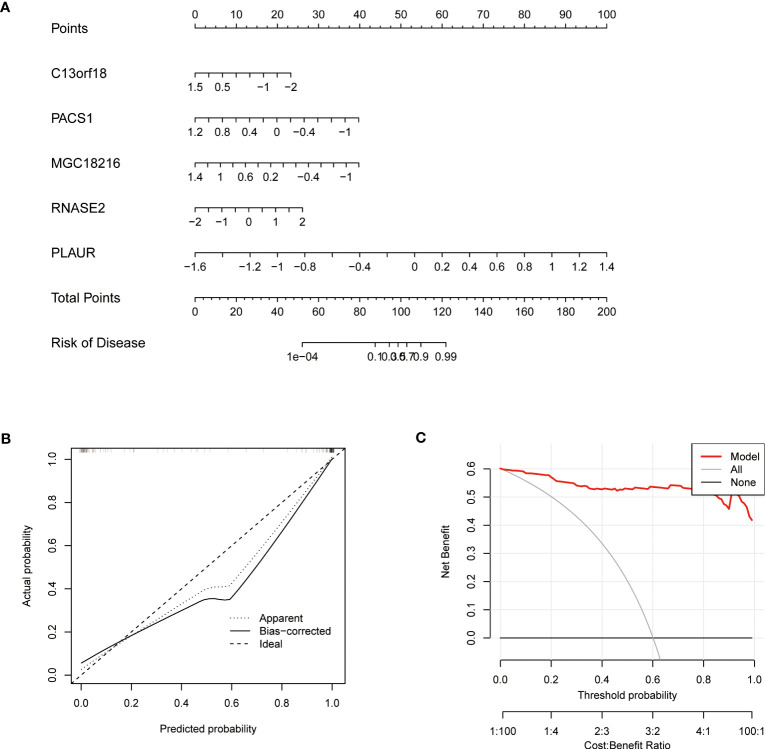
Validation of a machine learning model based on 5 genes for predicting TB Validation of the 5-gene-based XGB model. **(A)** Construction of a nomogram for predicting the risk of TB clusters based on the 5-gene-based XGB model (C13orf18, PACS1, MGC18216, RNASE2, and PLAUR). **(B)** Construction of the calibration curve. Calibration curve analysis exhibited that solid line was near the dotted line,which suggesting the accuracy of the nomogram was relatively high. **(C)** Construction of the decision curve. DCA exhibited that the red line moved away from the gray line, which suggesting the accuracy of the nomogram was relatively high.

A nomogram was constructed to estimate the risk of cuproptosis clusters in 92 TB patients (**Figure 9A**). The prediction performance of the nomogram was evaluated by calibration curves and DCA. The predicted outcomes were consistent with the actual outcomes (**Figure 9B**). DCA indicated that the accuracy of the nomogram was relatively high, providing a net clinical benefit (**Figure 9C**).

### Assessment of machine learning models

**Figure 10:**
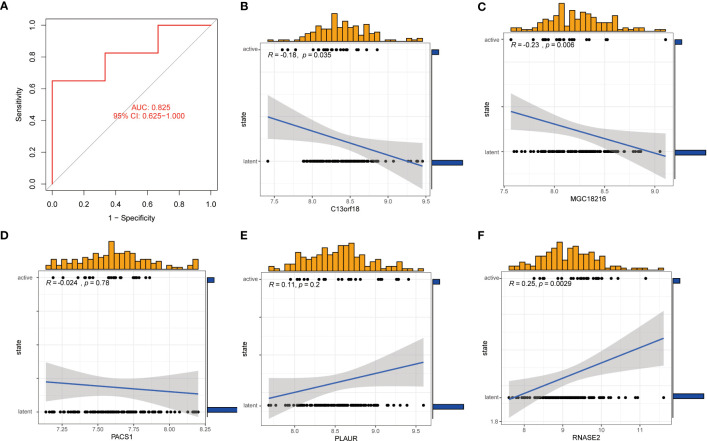
Correlation analysis between gene expression and disease status in an independent dataset of patients with TB. **(A)** the ROC curve of the five genes of the XGB model. the ROC curve of the five genes of the XGB model exhibited good performance (AUC= 0.825) **(B-F)** Correlation between the 5 genes and active/latent TB. C13orf18 and MGC18216 were negatively correlated with active TB. RNASE2 was positively correlated with active TB.

The GSE152532 dataset was utilized to validate the accuracy of the machine-learning model. In the GSE152532 dataset, the ROC curve of the five genes of the XGB model (C13orf18, PACS1, MGC18216, RNASE2, and PLAUR) exhibited good performance (AUC= 0.825) (**Figure 10A**). The TB patients in GSE152532 dataset could be divided into latent TB and active TB groups. Based on the clinical characteristics, the five genes were utilized to predict latent and active TB (**Figures 10B-F**), C13orf18 (R=-0.18) and MGC18216 (R=-0.23) were negatively correlated with active TB (**Figures 10B, C**). RNASE2 (R=0.25) was positively correlated with active TB (**Figure 10F**).

## Discussion

TB is mainly classified by the detection of Mtb, including AFB and culture of the pathogenic microorganism (2–4). However, there is an increasing consensus that the accuracy of AFB is not high (5–7). Although the culture of pathogenic microorganisms exhibits high specificity, false-positive cultures for Mtb are not rare (5, 8). Hence, comprehending the pathogenesis of TB and identifying the most appropriate molecular clusters of TB is crucial for enhancing the diagnosis and treatment of this patient population. A previous study suggested that dysregulation of copper homeostasis and cell death are involved in the pathogenesis of TB (15, 27). However, little is currently known about the role of cuproptosis, a novel cell death mechanism, in TB (17). Herein, we sought to elucidate the specific role of CRGs in the TB phenotype and immune microenvironment. Additionally, gene signatures related to cuproptosis were applied to predict the TB subtypes.

In this study, the expression profiles of CRGs were comprehensively analyzed in the blood of normal subjects and patients with TB for the first time. Compared with the normal population, 11 CRGs were abnormally expressed in patients with TB, including NFE2L2, NLRP3, ATP7B, SLC31A1, MTF1, DLD, LIAS, LIPT1, DLAT, GLS, and DBT, suggesting that CRGs play an essential role in the development of TB. There is a rich literature available substantiating that NFE2L2, NLRP3, and GLS genes may be involved in the pathogenesis of TB. Guiyi Ji et al. demonstrated that abnormal expression of NFE2L2 is associated with susceptibility to TB (28). Kai S. Beckwith et al. found that the plasma membrane was damaged by Mtb infection, which led to NLRP3 activation and pyrosis (29). Emerging evidence suggests that Mtb could cause damage to alveolar epithelial cells, while GLS is necessary for alveolar epithelial regeneration (30, 31). However, whether the other eight genes (ATP7B, SLC31A1, MTF1, DLD, LIAS, LIPT1, DLAT, and DBT) are involved in the pathogenesis of TB remains unclear. Subsequently, the correlation between CRGs was calculated to elucidate the relationship between the mutual regulation of CRGs and TB. Among the CRGs, the most significant correlations were found between NFE2L2 and MTF1, LIPT1 and DLAT, DLD and DLAT, and GLS and DBT. To our knowledge, no study has hitherto revealed the mutual regulatory mechanism of these genes in TB. Moreover, we found that the abundance of immune cells changed between the control and TB groups, consistent with findings reported by Hunter et al. (32). In this respect, we found that the levels of CD8+ T cells, follicular helper T cells, and resting memory CD4+ T cells were decreased in the TB group. Previous studies suggested that CD8+ T cells contribute to a protective immune response to Mycobacterium TB infection and that CD8+ T cell depletion may be a factor in susceptibility to TB (33, 34). Kumar et al. suggested that decreased abundance of follicular helper T cells was a characteristic of TB (35). We also found that the levels of monocytes, M0, M1, and M2 macrophages, activated dendritic cells, eosinophils, and neutrophils were increased in the TB group. Macrophages are responsible for implementing the cellular inherent antibacterial mechanism and initiating and maintaining inflammation, which plays a crucial role in the protection of the organism but also leads to TB progression (36).

Mtb can infect and incubate within macrophages, leading to the development of TB as infected macrophages proliferate (37, 38). Stimulated by antigenic peptides of intracellular pathogens, the abundance of M1 macrophages is increased to produce inflammatory cytokines, which can lead to more serious cell injury (39). Meanwhile, during extracellular pathogen invasion, Th2 cells/mast cells/basophils (or stimulation by IL-4, IL-10, IL-13, and immune complexes) can differentiate macrophages into the M2 state, which promotes intracellular TB infection (40, 41). The abundance of dendritic cells and neutrophils may be increased in the TB group (42, 43). The eosinophils are part of the granulocyte response in TB, which may also promote host drug resistance (44). In addition, we found that CRGs are associated with various immune cells in the TB group, but the underlying mechanism remains unclear, warranting further investigation.

Based on the expression profiles of 11 CRGs, a consensus clustering algorithm was utilized to cluster the 92 TB samples. It was found that there were significant differences between the two clusters. The expression levels of NFE2L2, NLRP3, SLC31A1, LIPT1, DLD, DLAT, MTF1, MTF1and DBT were significant increased in Cluster 2. In addition, the immune cell analysis shows that M0 macrophages, eosinophils, and neutrophils were significantly increased in Cluster 2, which suggested that Cluster 2 is characterized by immune cell activation and differentiation. These results suggested that the changes in these immune cells may be related to CRGs. The lipopolysaccharide (LPS) mediated regulation of myeloid cell differentiation and function was more active in TB patients of Cluster 1. LPS activation by pathogenic microorganisms can cause myeloid cell differentiation, activation of macrophages or(and) T cells, induction of systemic inflammatory responses, and even multiple organ function impairment (45–47). Meanwhile, we found that the protoporphyrinogen IX metabolic process was functionally active in TB patients of Cluster 1. However, the mechanism of the protoporphyrinogen IX metabolic process in TB remains unclear. KEGG pathway analysis showed that apoptosis and neurotrophin signaling pathways were significantly enriched in TB patients from Cluster 1. Stutz MD et al. verified that apoptosis plays an important role in the pathogenesis of TB (48). It has been reported that in patients with no brain infection, the lungs become infected with Mtb and cause an inflammatory response, which can trigger inflammation in the brain and interfere with neurotrophic factors (49). Interestingly, Cluster 2 was characterized by an active nervous system, suggesting that the nervous system of these patients may be affected by Mtb.

In recent years, machine learning models based on demographic and imaging metrics have been increasingly used to predict disease prevalence. Some studies confirmed that multivariate analysis takes into account the relationship between variables and therefore has a lower error rate and more reliable results than single-factor analysis (50, 51). Since TB is an infectious disease, there is an urgent need for accurate models to predict the prevalence of TB. This study compared the predictive performance of four machine learning classifiers (RF, SVM, GLM, XGB) based on the expression profiles of cluster-specific genes. The machine learning model of XGB yielded good performance in predicting TB subtypes. Subsequently, 5 important variables (C13orf18, PACS1, MGC18216, RNASE2, and PLAUR) were selected to construct the XGB model.

Studies have confirmed C13orf18’s involvement in the autophagy mechanism through its interaction with Beclin1, which contributes to the development of TB, suggesting that C13orf18 may participate in the pathogenesis of TB (52, 53). Pacs1 is a transport protein located in the cytoplasm, which plays a vital part in transporting calcium ions in the endoplasmic reticulum and supporting the development and survival of circulating lymphocytes. These functions suggest that Pacs1 could impact the advancement of TB by controlling the development and survival of lymphocytes, and hence, it could be a potential target for TB treatment, according to the literature (54–56). RNASE2, also known as an eosinophilic neurotoxin, belongs to the RNaseA superfamily and is one of the secreted proteins released after eosinophilic activation (57). When eosinophils are concentrated and activated at the site of Mtb infection, RNASE2 is released (57, 58). The uPAR protein encoded by PLAUR genes can regulate the regeneration of cells after different organs and represents a new molecular target for TB treatment (59, 60). However, the function of MGC18216 remains unclear and warrants further exploration.

The identification of active TB and latent TB is very crucial for clinical diagnosis and treatment (61). An individual with latent TB may test positive on immunological tests, but they do not exhibit any symptoms of the disease, and diagnostic tests, such as chest radiography, do not indicate any evidence of active TB (62). Nevertheless, weakened immunity can cause latent TB to become active TB at any point (63). The TST and IGRA are commonly applied to detect latent TB, but whether these methods can accurately identify latent TB remains unclear (64). Therefore, correlation analysis of latent and active TB was performed *via* the five predictive genes. We found that C13orf18 and MGC18216 exhibited a negative correlation with active TB, while RNASE2 positively correlated with active TB.C13orf18 can regulate phagocytes to promote phagolysosome formation or facilitate the accumulation of metals, thereby stimulating the microbial poisoning mechanism. Limiting the access of microorganisms to essential nutrients can help inhibit pathogenic microorganisms and lead to the onset of the latent phase of TB (52, 53, 65). The abundance of Mtb was increased during the active stage of TB infection, which led to the aggregation and activation of eosinophils, resulting in upregulated RNASE2 expression (57, 66). Taken together, the 5-gene-based XGB model exhibited satisfactory performance in evaluating TB subtypes and differentiating latent and active TB patients.

Some limitations of this research should be acknowledged. Firstly, the research was based on a comprehensive bioinformatics analysis, and further clinical or experimental evaluation should be necessary to verify the expression levels of CRGs. In addition, more clinical features are required to enhance the performance and robustness of the predicted model. Furthermore, refining the accuracy of cuproptosis-related clusters requires more TB samples. Finally, the potential correlation between CRGs and immune infiltration was not comprehensively investigated, emphasizing the need for more studies.

## Conclusion

Our study reveals an association between CRGs and infiltrating immune cells and illustrates significant immune heterogeneity between TB patients with different cuproptosis-related clusters. The XGB model based on 5 genes was the optimal machine learning model to evaluate the TB subtypes and differentiate latent and active TB patients. We provide hitherto undocumented evidence on the role of cuproptosis in TB and further elucidate the molecular mechanisms underlying immune heterogeneity in TB.

## Data availability statement

The original contributions presented in the study are included in the article/supplementary material. Further inquiries can be directed to the corresponding author.

## Author contributions

SL and QL designed the study, collected the original data and finished the analysis. LN and YZ drafted the initial manuscript. XM helped revise the manuscript. QZ provided the funding and supervised the study. The final manuscript was read and approved by all authors. All authors contributed to the article and approved the submitted version.
